# Comparision of the Cytotoxic Effects of Birch Bark Extract, Betulin and Betulinic Acid Towards Human Gastric Carcinoma and Pancreatic Carcinoma Drug-sensitive and Drug-Resistant Cell Lines

**DOI:** 10.3390/molecules14041639

**Published:** 2009-04-24

**Authors:** Marcin Drag, Pawel Surowiak, Malgorzata Drag-Zalesinska, Manfred Dietel, Hermann Lage, Józef Oleksyszyn

**Affiliations:** 1 Division of Medicinal Chemistry and Microbiology, Faculty of Chemistry, Wroclaw University of Technology, Wybrzeze Wyspianskiego 27, 50-370 Wroclaw, Poland; E-mail: jozef.oleksyszyn@pwr.wroc.pl (J.O.); 2 Department of Histology and Embryology, University School of Medicine, ul. Chalubinskiego 6a, 50-356 Wroclaw, Poland; E-mails: pawel.surowiak@interia.pl (P.S.), drag@hist.am.wroc.pl (M.D-Z.); 3 Charité Campus Mitte, Institute of Pathology, Chariteplatz. 1 20/21, D-10117 Berlin, Germany; E-mails: hermann.lage@charite.de(H.L.), manfred.dietel@charite.de (M.D.); 4 Lower Silesian Oncology Centre, pl. Hirszfelda 12, 53-413 Wroclaw, Poland

**Keywords:** Betulin, Betulinic acid, Birch, Gastric carcinoma, Pancreatic carcinoma

## Abstract

Betulin and betulinic acid are naturally occurring pentacyclic triterpenes showing cytotoxicity towards a number of cancer cell lines. These compounds can be found in the bark of the many plants. In this report we have compared the cytotoxic activity of crude birch bark extract and purified betulin and betulinic acid towards human gastric carcinoma (EPG85-257) and human pancreatic carcinoma (EPP85-181) drug-sensitive and drug-resistant (daunorubicin and mitoxantrone) cell lines. Our results show significant differences in sensitivity between cell lines depending on the compound used, and suggest that both betulin and betulinic acid can be considered as a promising leads in the treatment of cancer.

## 1. Introduction

Chemotherapy is a standard treatment for advanced neoplastic diseases, particularly following surgical removal of tumors. Considering the fact that the main aim of the chemotherapy is to eliminate remaining neoplastic cells from the organism, the success of this treatment determines the final result of the recovery process. One of the main reasons for unsuccessful chemotherapy outcomes is the resistance of the cancer cells to cytostatic or cytotoxic drugs. Many mechanisms are responsible for the resistance of cancer cells to cytostatics, among which the most recognized are expression of ABC-transporters, apoptosis mechanism disorders, DNA repair disorders, increased binding of the drugs in the cells, overexpression of protooncogenes, and decreased expression of tumor suppressors [1,2]. One of the well described multidrug resistance (MDR) ABC-transporters is P-glycoprotein (P-gp/MDR1). P-gp dependant resistance is called typical MDR. Resistance dependant on all the other ABC-transporters is called atypical MDR [2]. Several methods to overcome resistance to chemotherapeutics have been described, for example gene silencing using siRNA, rybozymes or viruses [3,4,5]. However, due to the parallel multiple factors responsible for drug resistance, to date these methods are not broadly considered practical. In recent years considerable attention has been paid to low molecular weight molecules that inhibit or suppress the cell growth and multiplication for the potential application to cancer therapy.

A promising group of such low molecular compounds are betulin and its oxidation product, betulinic acid. These compounds are found in birch bark and depending on the method of extraction, betulin usually comprises more than 90% of the total isolated products, and betulinic acids accounts for between 1-5%. The first report on betulinic acid by Pisha *et al*. described the anticancer activity of this compound toward human melanoma [6]. Later reports described betulinic acid as an agent active towards several different types of cancer and cancer cell lines such as ovarian carcinoma, neuroectodermal tumors (Ewing`s sarcoma, neuroblastoma, meduloblastoma), human leukemia HL-60, neck squamous cell carcinoma SCC25 and SCC9 or malignant brain tumors [7,8,9,10,11]. The broad activity of betulinic acid toward various cancer cells was recently reported by Kessler *et al*. [12]. However, some reports mention that betulinic acid failed to affect colon carcinoma, breast carcinoma, renal cell carcinoma, small cell lung carcinoma or T-cell leukemia [9]. Moreover, Zuco *et al*., observed that betulinic acid acts on tumor cell lines, but not on normal cells [13]. All these observations resulted in the incorporation of betulinic acid to the Rapid Access to Intervention Development (RAID) program of the U.S. NIH [14].

To date there are only a few reports comparing the effect of betulin derivatives on drug-sensitive and drug-resistant cell lines. For example Fernandes *et al.* described the inhibition of growth and induction of apoptosis of K562 cell line (erythroleukemia) in its drug-sensitive and vincristine (Lucena 1) drug-resistant cell lines [15]. Jung *et al.* described cytotoxicity of betulinic acid toward both parental and drug resistant (5-fluorouracil and oxaliplatin) colon cancer SNU-C5 cell lines [16]. Such an effect was not observed in cells resistant to irrinotecan. However, these authors focused on the sensitization of cell lines to betulinic acid but did not report any specific data on the effect of betulinic acid toward these cell lines. Also Fulda and Debatin and Sawada *et al.* described sensitization of multiple cell lines to chemotherapy [17,18]. We have also very recently found that betulinic acid displays almost equal cytotoxicity toward drug-resistant (cisplatin, etoposide, vinblastin and fotemusine - MeWo CIS, MeWo ETO, MeWo VIN and MeWo FOTE) and drug-sensitive melanoma cell lines (MeWo) and additionally reveal stronger cytotoxic activity toward normal melanocyte cell line (NHEM-neo) [19].

The mechanism of action of betulin derivatives is not clear, although Fulda *et al*. have suggested that betulinic acid induces loss of mitochondrial membrane potential and the release of the pro-apoptotic proteins cytochrome c and Smac [9]. However, it has also been proposed that betulinic acid kills prostate cancer cells via activation of selective proteasome-dependent degradation of transcription factors, specificity protein 1 (Sp1), Sp3, and Sp4 [20].

To date, the majority of studies were devoted to the investigation of betulinic acid, as this derivative revealed better bioavailability and cytotoxic effects. However, the need for chemical transformation of betulin into betulinic acid makes the more former compound an interesting medical target, especially because it is also a known natural medicinal product. Betulin and its derivatives were reported to be less active toward some cell lines, but no particular structure activity studies were performed to explain in detail the difference in the activity. One possibility could be much lower solubility of betulin comparing to betulinic acid due to the presence of the much more hydrophilic carboxylic group in the latter, which may adversely affect cell permeability and cell penetration in the case of the betulin [21]. Additionally, there are some very limited data on the cytotoxicity of crude birch bark extract toward cancer cell lines.

The aim of this work was to compare the cytotoxicity of a crude extract of birch bark, pure betulin, and betulinic acid toward drug-sensitive and drug-resistant human gastric carcinoma (EPG85-257) and human pancreatic carcinoma (EPP85-181) cell lines. The selection of the tumor models was based on two criteria. First, the potential route of drug administration (oral), which allows delivery of sufficient amount of the active ingredient to the target, especially in the case of gastric carcinoma. The second criteria was the extreme resistance of both types of carcinoma to any pharmaceutical therapy in clinical practice. To allow reliable interpretation of the data in this work we have investigated these well defined models of typical and atypical MDR [22,23,24,25,26].

## 2. Results and Discussion

### 2.1. Quantitative and qualitative analysis of betulin, betulinic acid, and birch bark extract

**Figure 1:**
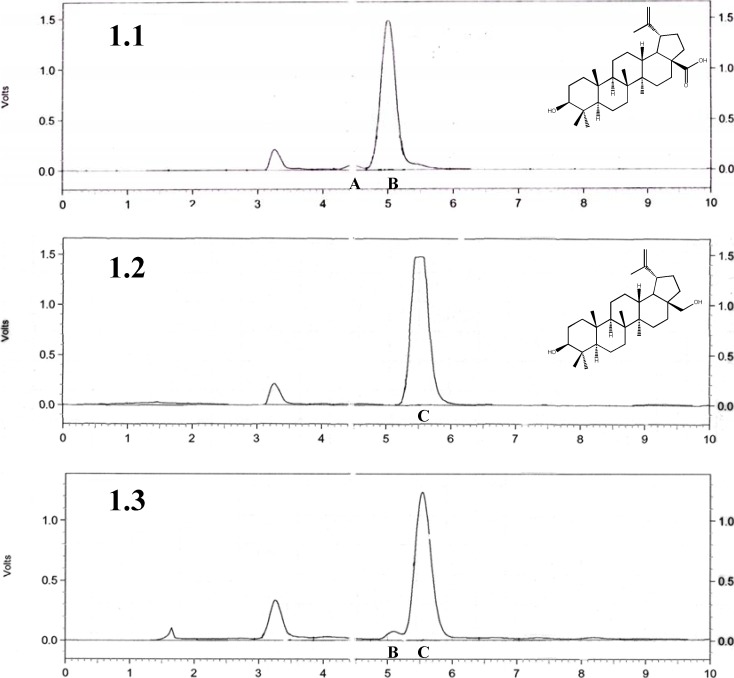
HPLC analysis: 1.1 betulinic acid, **A** epimer α and **B** epimer β of the compound; 1.2 betulin chromatogram, **C** betulin; 1.3 birch bark extract, **B** epimer β of the betulinic acid, **C** betulin.

We used a procedure employing ethanol as solvent for the isolation of the betulin and betulinic acid from birch bark, since this method allows for almost complete isolation of both terpenes from solid phases. HPLC analysis (Figure 1C) revealed that the isolated fractions comprised around 91% of the betulin and 4% of the betulinic acid. Other components are undetectable using HPLC. This result is comparable to those obtained in a previous report on the isolation and HPLC analysis of the ethanolic fraction of birch bark by Zhao *et al*. [27]. The ^1^H-NMR analysis confirms earlier results and shows dominating characteristic resonance signals coming from betulin and in some cases betulinic acid (Suppl. Material) [28]. The aliphatic fragment of the spectra (0.5-1.7 ppm) indicates the presence of some additional compounds (calculated by the total amount of the protons comparing to the betulin spectra), but the complex character of these spectra in this case makes any predictions about the structure or even the character of the other compound(s) impossible. HPLC analysis of the purified betulin (Figure 1B) reveals that this compound was more than 95%+ pure. This was also confirmed by ^1^H-NMR spectra (Suppl. Material). Betulinic acid obtained by oxidation of betulin via betulonic acid using the method described by Kim (Figure 1A) shows the presence of the dominating β form to around 96% and some of the α form of this compound [28].

The overall purity of combined forms of betulinic acid obtained in this way was more than 95%, as additionally confirmed by ^1^H-NMR analysis (Suppl. Material). These results indicate that, in the case of the birch bark extract, the predominating compound is betulin. However, an interesting observation was made during preparation of the DMSO solutions of all three compounds for the biological studies. The birch bark extract had excellent solubility even at very high milimolar concentrations, whereas pure betulin even in the low milimolar concentrations was barely soluble and warming was required to achieve the desired clarity of the sample. Betulinic acid was much more soluble than betulin, but worse compared to the birch bark extract. This observation intrigued us and suggested that the birch bark extract contains additional components detectable neither by HPLC nor analysis that improve the solubility of the predominating betulin. 

**Figure 2:**
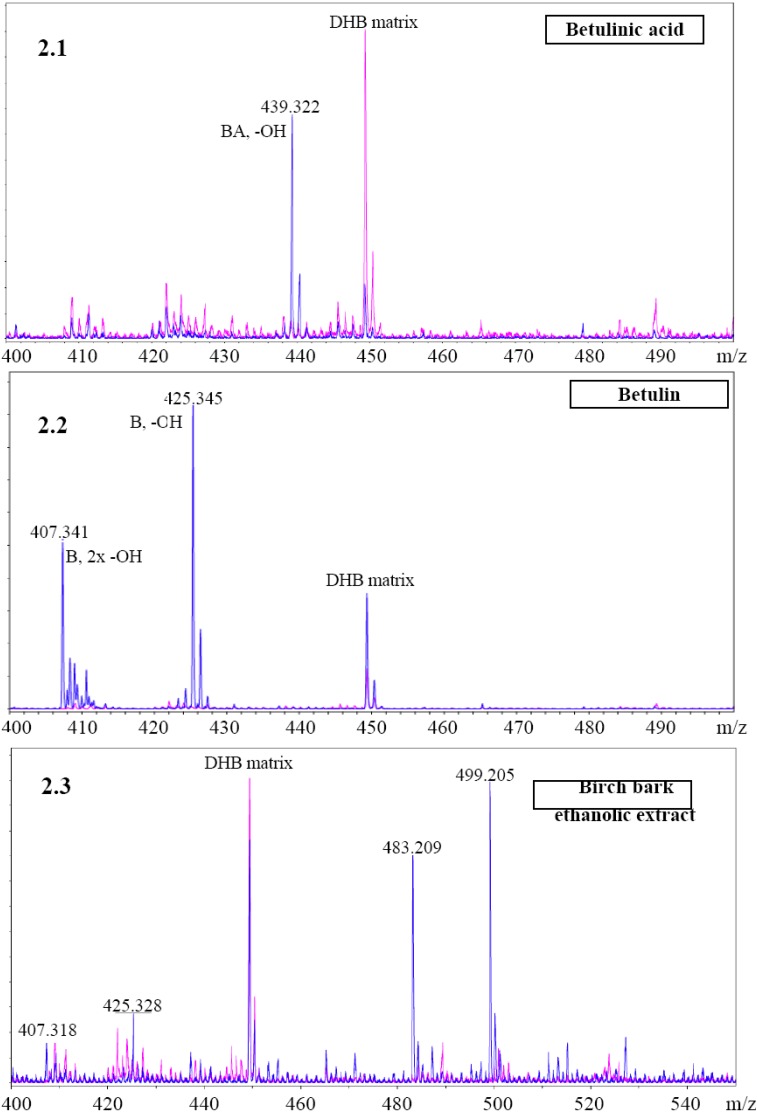
(**A**) MALDI mass spectroscopy analysis: (**2.1**) betulinic acid; (**2.2**) betulin; (**2.3**) birch bark extract; DHB-2,5-dihydroxybenzoic acid matrix.

In the MS the molecular masses of betulinic acid (Figure 2A, signal at 439.322, betulinic acid mass minus one hydroxyl group) and betulin (Figure 2B, two signals at 425.345 and 407.341 representing betulin mass minus one and two hydroxyl group(s) respectively) could be observed, while in the case of the birch bark extract we can see minor signals coming from free betulin (Figure 2C, 425.328 and 407.318) and two predominating signals at 499.205 and 483.209 mass units suggesting some additional compounds. Unfortunately, despite this and several additional attempts we were unsuccessful in identification of this molecule(s).

### 2.2. Comparison of the anticancer activity of the betulin, betulinic acid and birch bark extract

**Figure 3:**
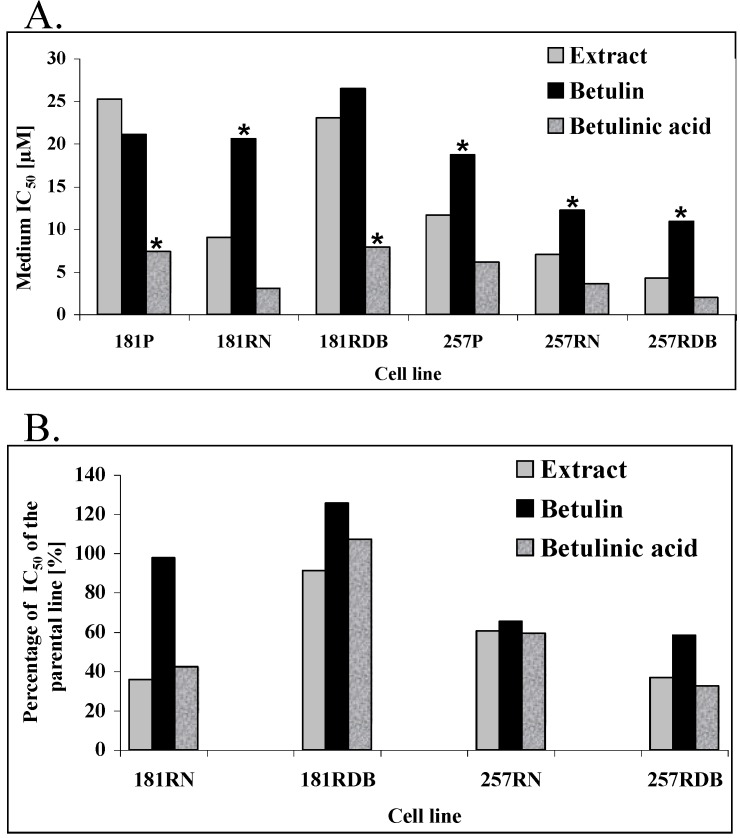
A: IC_50_ of the tested compounds. B: Relative IC_50_ values of the tested compounds for the drug resistant cell-lines compared to the parental lines (181RNOV and 181RDB compared to 181P, 257RNOV and 257RDB compared to 257P).

Using the SRB method we investigated the influence of betulin, betulinic acid and birch bark extract on drug-sensitive and drug-resistant cell lines. IC_50_ values for the individual substances are presented in Table 1 and Figure 3A. Analysis of the results using the ANOVA rank test of Kruskal-Wallis revealed that in the case of the human pancreatic carcinoma lines 181P, 181RDB, and 181RN, betulinic acid is significantly better compared to the other two substances. The IC_50_ values for betulin were in the range of 21.09 to 26.5 μM and for the birch bark extract between 9.07 to 25.26 μM, whereas for betulinic acid these values were between 3.13 and 7.96 μM. Interestingly, in the case of the Mitoxantrone resistant 181RN cell line, the extract from birch bark and betulinic acid are around three times more active compared both to betulin and to the remaining two other human pancreatic carcinoma cell lines (Figure 3B).

* indicates significantly different values. IC50 (P < 0.05).

In the case of the human gastric carcinoma cell lines 257P, 257RNOV, and 257RDB, the IC_50_ for the betulin is significantly higher compared to betulin and betulinic acid (Table 1, Figure 3) being in the range 10.97 to 18.74 μM. IC_50_ values for betulinic acid (2.01 - 6.16 μM) are around two times lower compared to the birch bark extract values (4.29 - 11.66 μM).

**Table 1:** IC_50 _values of the tested compounds towards parental lines and their drug-resistant sublines. Results obtained using the ANOVA rank test of Kruskal-Wallis. Comparison between the substances and their IC_50_ towards parental lines and their drug resistant sublines.

Studied cell line	Betulin IC_50_ [μM]	Extract IC_50_ [μM]	Betulinic acid IC_50_ [μM]	Comparison between substances P value
181P	21.09	25.26	7.42	0.0182
181RNOV	20.62	9.07	3.13	0.0182
181RDB	26.50	23.03	7.96	0.0186
Comparison between parental and resistant lines P value	0.0273	0.0273	0.0665	
257P	18.74	11.66	6.16	0.0062
257RNOV	12.25	7.08	3.66	0.0073
257RDB	10.97	4.29	2.01	0.0069
Comparison between parental and resistant lines P value	0.0273	0.0273	0.0273	

**Figure 4:**
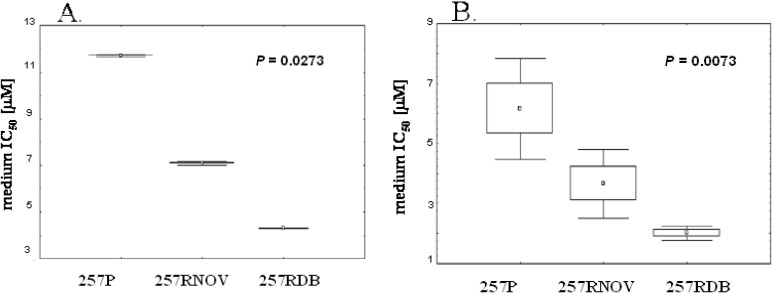
**(A)** Statistically significant differences of IC_50_ values toward 257P, 257RNOV and 257RDB cell lines for the birch bark extract (**A**.) and betulinic acid (**B**.)

Interestingly, both drug-resistant human pancreatic carcinoma cell lines are 2-3 times more sensitive to treatment with all the three substances (Figure 3B). The biggest difference is observed when comparing the parental cell line (257P) with the daunorubicin resistant cell line (257RDB), where the birch bark extract and betulinic acid are around three times more effective for the latter. Figure 4 shows a comparison of IC_50_ values for all human gastric carcinoma (257P) cell lines from three independent experiments for the birch bark extract (Figure 4A) and for betulinic acid (Figure 4B), clearly indicating these differences.

## 3. Experimental

### 3.1. Materials and methods

All the chemicals and solvents were obtained from commercial suppliers and used without further purification, unless otherwise stated. Column chromatography was performed using grade 60 silica gel (Fisher Scientific, 70–230 mesh). ^1^H-NMR spectra were obtained with the aid of the Burnham Structural Biology facility using a Varian 300 spectrometer in chloroform-d_3_ or dimethyl sulfoxide-d_6_ (Aldrich). Analytical high performance liquid chromatography (HPLC) analysis were conducted on a Beckman-Coulter System Gold 125 solvent delivery module equipped with a Beckman-Coulter System Gold 166 Detector system by using a Varian Microsorb-MV C18 (250 x 4.8 mm) column. Mass spectra were recorded in MALDI mode with the aid of the Burnham Proteomics facility.

### 3.2. Plant material

The white birch (*Betula pendula* Roth, syn. *Betula verrucosa* – European White Birch - (Betulaceae) was authenticated by corresponding author using http://www.atlas-roslin.pl/gatunki/Betula_pendula.htm) bark samples (around 10 different trees) were collected in summer 2004 from the forest near Wroclaw (Lower Silesia) in Poland. All the collected birch barks were mixed equally and dried at room temperature in a dark and dry place for a week.

### 3.3. Preparation of the birch bark extract

The white bark (approx. 250 g) was placed in a 3 liter round bottomed flask and refluxed in anhydrous ethanol (100%, 1.5 liter) for 4 hours. The remaining solid material was filtered off and the yellowish solution was evaporated to dryness using a rotary evaporator with a bath temperature of 45°C. The resulting yellowish powder was additionally dried in 40°C on a glass plate in a dark chamber overnight (yield 66 g, 26.4%). The samples were stored at room temperature in the dark. Dry samples of the birch bark extracted using ethanol were used in further biological experiments.

### 3.4. Purification of the betulin from the extract

The sample (2 g) of the birch bark extract was purified using column chromatography (glass column filled with grade-60 silica gel (70–230 mesh, 110 cm in length, 4 cm in diameter) using a mixture of chloroform/ethyl acetate solvents in the ratio 80:20. The fractions containing betulin were combined and evaporated using a rotary evaporator. Betulin was oxidized to betulinic acid as previously described [28]. All the samples were dissolved in histological grade (Sigma-Aldrich) DMSO and were stored at the -20°C.

### 3.5. HPLC analysis

Analytical high performance liquid chromatography (HPLC) analysis was conducted on a Beckman-Coulter System Gold 125 solvent delivery module equipped with a Beckman-Coulter System Gold 166 Detector system by using a Varian Microsorb-MV C18 (250 x 4.8 mm) column (La Jolla, CA). 1mg of the birch extract, betulin or betulinic acid was dissolved in 1 mL of the methanol and 20 μL was injected into the column. Analysis was performed for 10 min using as mobile phase acetonitrile–water in the ratio 78:22. UV detector was set at *λ* = 210 nm.

### 3.6. NMR analysis

^1^H-NMR spectra were obtained with the aid of the Burnham Structural Biology Facility (La Jolla, CA) using a Varian 300 spectrometer in chloroform-d_3_ (betulin and birch extract) or dimethyl sulfoxide-d_6_ (betulinic acid). ^1^H-NMR (300 MHz) spectra are reported as follows: chemical shifts in ppm downfield from TMS, the internal standard. In every experiment, 1 mg of the birch extract, betulin and betulinic acid was dissolved in 0.7 mL of the appropriate solvent and subjected to the NMR analysis.

### 3.7. Mass spectroscopy analysis

Mass spectra were recorded with the aid of the Burnham Proteomics Facility (La Jolla, CA) using MALDI/TOF. Experiments were performed using a DHB (2,5-dihydroxybenzoic acid) matrix.

### 3.8. Cell lines

Characteristics and culture of the human gastric carcinoma cell line EPG85-257P (257P) and human pancreatic cell line EPP85-181P (181P), its classical MDR variants EPG85-257RDB (257RDB) and EPP85-181RDB (181RDB) overexpressing MDR1/P-gp, and its atypical MDR variants EPG85-257RNOV (257RNOV) and EPP85-181RNOV (191RNOV) were described in detail previously (Table 2).

**Table 2:** Cancer cell lines with drug-resistant sub-lines.

Cell line	Origin	Selection agent	Supposed resistance mechanisms
EPP85-181P	Pancreatic carcinoma		
EPP85-181RNOV		Mitoxantrone	Topo II
EPP85-181RDB		Daunorubicin	MDR1/P-gp
EPG85-257P	Gastric carcinoma		
EPG85-257RNOV		Mitoxantrone	BCRP, GPC3, Topo II, TAP
EPG85-257RDB		Daunorubicin	MDR1/P-gp

### 3.9. Cell culture

Cells were grown in Leibovitz L-15 medium (Biowhittaker, Walkersville, MD) supplemented by 10% fetal calf serum (FCS) (Gibco/BRL, Grand Island, NY), 1 mM L-glutamine, 6.25 mg/L fetuin, 80 IE/L insulin, 2.5 mg/mL transferrin, 0.5 g/L glucose, 1.1 g/L NaHCO_3_, 1% minimal essential vitamins and 20,000 kIE/L trasylol in a humidified atmosphere of 5% CO_2_ at 37°C.

### 3.10. Cell proliferation assay

Chemoresistance was tested using a proliferation assay based on sulphorhodamine B (SRB) staining as described previously [26]. Briefly, 800 cells per well were seeded in triplicate in 96-well plates. After attachment for 24 h, substances were added in dilution series for a 5-day incubation, before SRB staining was performed. Incubation was terminated by replacing the medium with 10% trichloroacetic acid, followed by further incubation at 4°C for 1h. Subsequently, the plates were washed five times with water and stained by adding 100 µL 0.4% SRB (Sigma, St. Louis, MO,USA) in 1% acetic acid for 10 min at room temperature. Washing the plates five times with 1% acetic acid eliminated unbound dye. After air-drying and re-solubization of the protein bound dye in 10 mM Tris-HCl (pH=8.0) absorbance was read at 562 nm in an Elisa-Reader (EL 340 Microplate Bio Kinetics Reader, BIO-TEK Instruments, Winooski, VT, USA). The measurements were performed in triplicate in three independent experiments. IC_50_-values were calculated from three independent experiments for each cell line.

### 3.11. Statistical analysis

Statistical analysis of the results took advantage of Statistica 98 PL software (Statsoft, Krakow, Poland). *P* < 0.05 was considered to indicate a significant relationship.

## 4. Conclusions

Low molecular weight molecules are vital weapons in the fight against different kinds of tumors. However, some types of cancer are more resistant to chemotherapy, and their diagnosis in the advanced clinical stages gives bad survival prognostics for the patient. Natural compounds are promising tool in the treatment of the several types of cancer and several of them are in clinical trials. A group of such compounds are triterpenes isolated from the birch bark.

In this report we show that betulin and its derivative betulinic acid reveal cytotoxicity towards human gastric carcinoma (EPG85-257) and human pancreatic carcinoma (EPP85-181) drug-sensitive and drug-resistant cell lines. All the tested compounds revealed significant cytotoxicity, with the most effective being betulinic acid and the extract from birch bark. On the basis of published results, one could expect activity for the betulinic acid, but quite surprisingly the birch bark extract of bark was only slightly less effective, especially in the case of human gastric carcinoma cell lines. Our chemical analysis revealed only small amounts of betulinic acid (about 4%) in the extract, thus we can assume that the contribution of this compound to the observed overall cytotoxic effect is rather minor. On the other hand, purified betulin was in almost every case the least active out of the three tested compounds.

Another interesting observation from our studies is the higher activity of the birch bark extract and betulinic acid toward drug-resistant cell lines, especially in the case of human gastric carcinoma. These models have well defined resistance mechanism via overexpression of the ABC-transporters. According to published data, the mechanism of action of betulinic acid via apoptosis, a conserved pathway not known to be altered in the tested here cell lines. However, one should be careful when judging the mechanism of action of the tested compounds, because apoptosis pathways are controlled by multiple factors, and commonly performed assays are not always indicative and can be misleading as recently shown for betulinic acid and others low molecular weight molecules considered as inductors of apoptosis [29,30].

Finally, the drug-resistant models examined here are extremely resistant to the chemotherapy. Our results shows that the molecules tested here are capable of inducing a promising cytotoxicity level, whereby the method of delivery of drugs for these types of cancers (preferable oral) can help to achieve the desired dosage at satisfactory levels, not only for betulinic acid but even for the naturally occurring betulin, which is easily available in almost unlimited amounts.
